# Analyzing the Effectiveness of Macroeconomic Policies in Addressing Poverty in Iraq: An Econometric Study considering Digital Transformation and Artificial Intelligence for the Period (2004–2024)

**DOI:** 10.12688/f1000research.174407.1

**Published:** 2026-02-06

**Authors:** Nadhem Abdullah Abid Al Mihimdy, Ahmed Abid Saleh Attia, Ministry of Finance–General Tax Authority

**Affiliations:** 1 Al-Huda University College, alnbar, bgdad, Iraq; 2 Ministry of Finance–General Tax Authority, alnbar, bgdad, Iraq

**Keywords:** Macroeconomic Policies, Absolute Poverty Line, ARDL Methodology, Digital Transformation, Artificial Intelligence.

## Abstract

**Background:**

Digital transformation and artificial intelligence (AI) have become increasingly important in enhancing the effectiveness of macroeconomic policies, particularly through improved economic data management and more efficient targeting of vulnerable groups. Poverty reduction remains one of the most critical challenges for policymakers in Iraq due to its wide economic and social implications. Addressing this challenge requires a comprehensive mix of macroeconomic policies tailored to the national context. This study examines the effectiveness of key macroeconomic policy instruments in reducing poverty in Iraq during the period (2004–2024).

**Methods:**

The Autoregressive Distributed Lag (ARDL) model is employed, along with the Bounds Testing approach, to investigate the existence of a long-run cointegration relationship between the absolute poverty line and major macroeconomic policy variables, including broad money supply, inflation rate, government expenditure, and per capita foreign trade. Annual time-series data for the period were analyzed to estimate both short-run and long-run dynamics.

**Results:**

The results indicate a statistically significant long-run equilibrium relationship at the (1%) level between macroeconomic policy instruments and the absolute poverty line. The empirical findings show that monetary policy exerts the strongest influence on poverty levels, with broad money supply, inflation, and per capita foreign trade displaying significant effects. Government expenditure plays a meaningful role in reducing poverty in both the short and long run.

**Conclusions:**

The study concludes that integrating digital transformation and AI technologies into the design and implementation of macroeconomic policies can substantially enhance their effectiveness in reducing poverty. Key recommendations include establishing a National Council for Digital Transformation and Artificial Intelligence within the Ministry of Planning, strengthening digital infrastructure through public–private partnerships, activating electronic interlinkages among ministries via unified databases, enhancing financial inclusion platforms targeting vulnerable groups, improving government spending efficiency, and encouraging investments that generate sustainable employment opportunities.

## Introduction

Poverty is one of the most prominent challenges facing Iraq at the economic and social levels since 2003, as it reflects the weak purchasing power of households, the uneven distribution of income and the absence of economic justice. There is a need to adopt effective strategies that promote economic activity, improve living standards, provide employment opportunities and basic services to reduce social inequality and achieve sustainable economic stability. Confronting poverty requires an integrated vision that combines wise management of resources, rationalization of economic and social policies, and the activation of inclusive growth mechanisms that ensure a fair distribution of wealth and promote societal justice. Leveraging modern technologies for digital transformation and artificial intelligence to enhance the efficiency of macroeconomic policies reflects the country’s ability to create a stable and prosperous economic and social environment.

### Research problem

Despite the adoption of various macroeconomic policies in Iraq since 2004, poverty rates are still high and its roots have not been addressed sustainably, as the rapid decline of poverty indicators appears in the years of oil abundance and returns to rise with any economic or financial crisis.

How effective are the macroeconomic policies adopted in Iraq during the period (2004-2024) in addressing poverty and reducing its effects?

### Importance of research

The importance of the research stems from the fact that:

It seeks to analytically and quantitatively evaluate macroeconomic policies in Iraq over two decades, contributes to revealing the extent to which fiscal, monetary, and trade policies have succeeded in reducing poverty rates compared to social protection programs, and provides a knowledge framework for decision-makers to develop more effective policies in distributing resources and strengthening social safety nets.

### Research hypotheses

The research seeks to test the following hypotheses:

1. The existence of a long-term equilibrium relationship (mutual integration) that moves from macroeconomic policy tools (monetary, fiscal, and trade) towards the level of poverty in Iraq, so that these policies have a direct impact on poverty reduction during the period (2004-2024).
2. Monetary policy is the most effective in influencing poverty levels in Iraq.

### Research objectives

The research aims to:

1. Measuring and analyzing the effectiveness of macroeconomic policies in addressing poverty in Iraq and testing the long-term equilibrium relationship and the speed of reaching the state of social and economic stability using the modern methodology of joint integration according to the (ARDL) model.
2. Providing practical recommendations that contribute to strengthening the role of digital transformation and artificial intelligence technologies in formulating macroeconomic policies to achieve social justice and address poverty.

### Research methodology

The research adopted a blended approach between the deductive and inductive methods to measure and analyze the effectiveness of macroeconomic policies in addressing poverty in Iraq in light of digital transformation and artificial intelligence, and used the quantitative econometric approach through the application of the statistical program (EViews 10) for estimation and for conducting the necessary econometric and statistical tests.

### Research limits

1. Spatial Limits: The Iraqi Economy.
2. Time Limits: The research covers the time period (2004-2024).

### Research structure

The research is divided into three main axis, preceded by an introduction. The first axis included the theoretical literature on the effectiveness of macroeconomic policies, poverty, digital transformation and artificial intelligence, while the second axis was concerned with standard modeling to analyze the effectiveness of macroeconomic policies in addressing poverty in Iraq for the period (2004-2024), while the third axis included the most important conclusions and recommendations.

## The first axis

### Theoretical literature on the effectiveness of macroeconomic policies, poverty, digital transformation and artificial intelligence

The theoretical literature on the effectiveness of macroeconomic policies constitutes a framework for understanding the ability of government interventions to address economic and social imbalances, highlighting poverty and its indicators as essential tools for measuring the results of these policies and the extent to which they are reflected on improving living standards. This framework is becoming increasingly important in light of technological advancements, as digital transformation and artificial intelligence are modern tools that can be employed to enhance the efficiency of these policies and increase their ability to respond to economic and social challenges.

**1-1: The Theoretical Framework for the Effectiveness of Macroeconomic Policies and Their Tools:**

Economic policies are defined as a set of measures, procedures, laws and positions set by the government, which reflect its perception and philosophy on how to manage economic resources and embody its various goals in the desired direction internally within the country’s economy. **( Aday, 2025: 123)**, and the most important of these policies are the following:

1. **Fiscal Policy:**

It is the main economic tool adopted by the state to manage public resources and revenues and direct expenditures in a way that achieves a balance between the goals of economic stability and the requirements of development, as it represents the framework through which it exercises the function of government intervention in economic activity through its tax and spending tools in order to address structural imbalances, stimulate growth and reduce poverty. Their effectiveness lies in their ability to coordinate with other macroeconomic policies such as monetary and trade policies to ensure the efficient distribution of resources and their fair use in serving the strategic objectives of the national economy **(** **Mawarid & Moussa, 2013** **: 313).**

The focus was on the variable of government spending, as it is one of the most important tools of fiscal policy that affects the standard of living of individuals and thus poverty rates, as increasing government spending on the productive and service sectors is supposed to contribute to providing job opportunities and improving public services, which leads to reducing poverty indicators **(** **Enyim, 2013** **: 5).**

2. **Monetary Policy:**

It is the main economic tool used by the monetary authorities, especially the Central Bank, to manage the money supply, direct interest rates, and regulate liquidity in the economy with the aim of achieving financial and monetary stability, and is based on clear theoretical foundations that define the relationship between money, inflation, economic growth and employment, as it works to control inflation rates, support investment, and promote sustainable growth Its importance lies in its ability to adapt to economic changes and coordinate with fiscal and trade policy to ensure the efficient distribution of resources and achieve the goals of economic development and macro-stability **(** **Fawaz, 2021** **: 92).**

The broad money supply ( **M2**) and the inflation rate are emphasized as monetary policy tools that affect the level of poverty, as an increase in the broad money supply usually leads to an increase in liquidity in the economy and stimulates economic activity, which may reduce poverty if accompanied by real growth, while an oversupply of money may lead to a rise in prices. The inflation rate directly affects the purchasing power of individuals, as the higher the inflation, the lower the capacity Purchasing which increases poverty rates and weakens the standard of living.

3. **Trade Policy:**

It is the set of procedures and strategies followed by the State to regulate foreign trade, whether through exports or imports, with the aim of achieving economic goals such as increasing growth, supporting productive sectors, protecting local industries, and improving the balance of payments, and its effectiveness reflects its ability to influence the movement of goods, services and capital, regulate competition, and ensure the integration of the national economy with global markets while maintaining macro-stability and achieving sustainable development by achieving a balance between local interests and the requirements of the international economy **(** **Adaika & Bouzid, 2018** **: 260).**

The average foreign trade per capita variable, which is one of the most important indicators used to measure the extent of the integration of the national economy in international trade, regardless of the restrictions imposed on it, was used, and this index represents the average of the per capita income of the country’s foreign trade, reflecting the relative importance of trade in the national economy. Poverty rates while the decrease in per capita reflects the limited benefit from trade and increases the severity of poverty and is calculated according to the following formula **(** **Al-Shabibi, 2008** **: 54):**

$$\text{Average foreign trade}\;\mathrm{per}\;\text{capita}=\frac{\text{Exports}+\text{Imports}}{\text{Population}}*1000$$ (1)

**1-2: The Theoretical Framework of Poverty:**

Poverty is one of the most prominent economic and social challenges, as it is not limited to low income, but extends to include multiple dimensions such as education, health, and standard of living, and these dimensions are measured through quantitative and qualitative indicators that reflect the level of economic well-being and the extent of equity in the distribution of resources within society.

**First: The concept of poverty:** Poverty is defined as a state of inability to meet the basic needs of food, housing, health, and education, **(** **Ephilip and Martine, 2014** **: 7),** and the poverty rate is not just a lack of income as is sometimes imagined, but it is a broad, multidimensional concept, and economists and researchers usually identify several dimensions of poverty so that its measurement and analysis is more accurate, and the most important dimensions of poverty are the following **(** **Youssef and Mahmoud, 2025** **: 6)** and **(** **Ahmed & Ali, 2024** **: 781):**

1. **Economic dimension (income and consumption):** It relates to the level of income available to an individual or family, and the extent to which it is sufficient to cover basic needs such as food, housing, and clothing, and is often measured in the poverty line (absolute or relative).
2. **Social dimension:** It includes poor education, illiteracy, lack of skills, and lack of social support networks and illustrates how poverty leads to marginalization and poor social inclusion.
3. **Health dimension:** Malnutrition, lack of access to health services, high rates of morbidity and mortality and health are key factors because their vulnerability exacerbates poverty and vice versa.
4. **The human dimension**: Poverty is seen here as a denial of basic abilities such as freedom of choice, participation in decision-making, and the ability to achieve a decent life.
5. **Geospatial dimension:** Poverty varies between rural and urban areas or between specific regions within a country and focuses on the unequal distribution of infrastructure and services.
6. **The political-institutional dimension is represented:** By poor political participation, lack of justice, poor transparency, and discrimination that limits the opportunities of the poor.
7. **Psycho-cultural dimension:** Feelings of helplessness, marginalization, lack of self-confidence, and loss of hope for the future These intangible aspects make poverty deeper and more difficult to address.

**Second: Types of Poverty:** There are many types of poverty according to the angle of view (economic, social, temporal or geographical), the most important of which are the following:

1. **Absolute poverty:** It means the inability of an individual or family to meet the basic needs of life (food, housing, clothing, health, education) and is usually measured in the international poverty line (e.g., $2.15 per day according to the World Bank 2022) ( **Mustafa, 2002** **: 146).**
2. **Relative Poverty:** It is measured by comparing the income level of an individual or family with the average general income in society and means that an individual may have enough to survive but is considered poor because it is much lower than the prevailing level in his country **(** **Al-Khafaji, 2009** **: 6).**
3. **Extreme poverty:** A more severe level of absolute poverty, where an individual cannot even get enough food to survive **(** **United Nation, 2010** **: 1).**
4. **Multidimensional Poverty:** It is not limited to income only, but includes health, education, and standard of living, and is measured by the Multidimensional Poverty Index (MPI).
5. **Structural Poverty:** Poverty resulting from long-term economic and social imbalances such as chronic unemployment, poor infrastructure, and dependence on a single resource.
6. **Cyclical or situational poverty:** Caused by temporary crises or disasters (e.g. natural disasters, economic crises, wars) the situation may improve after the cause has disappeared.
7. **Urban poverty:** It appears in major cities because of unemployment, informal housing, high cost of living, pollution, and the absence of basic services.
8. **Rural poverty:** It is concentrated in rural areas due to poor infrastructure, lack of job opportunities, and dependence on traditional agriculture.

**Third: Economic Causes and Effects of Poverty in Iraq:** Poverty in Iraq is a complex phenomenon in which political, security, economic and social factors intertwine, making it one of the most prominent obstacles to sustainable development, as dependence on oil, weak economic diversification, administrative corruption and unemployment have contributed to deepening the roots of this problem, and this has negatively reflected on economic growth and productivity and increased the financial and social burdens on the state and members of society.

1. **Causes of poverty in Iraq:** The causes of poverty in Iraq are multiple, ranging from political and security factors related to instability and wars, and economic factors resulting from excessive dependence on oil and poor production diversification, as well as unemployment, administrative corruption, and social imbalances have contributed to the consolidation of this phenomenon and deepening its effects.
  a) **Political and security reasons:** They are represented in the repeated wars and the accompanying destruction of infrastructure, political instability, frequent changes of governments and policies, administrative and financial corruption that led to the waste of resources, as well as terrorism and internal displacement that deprived many families of their sources of livelihood **(** **Al-Yasiri, 2019** **: 311).**
  b) **Social causes:** They are represented in the high rates of unemployment, especially among youth and women, the decline in the level of education, the dropout of schools, the weakness of health services, the spread of chronic diseases, the disparity in income distribution, the absence of social justice, and the high rates of population growth without sufficient job opportunities **(** **Mawarid and Moussa, 2013** **: 308).**
  c) **Economic Reasons:** These reasons are over-dependence on oil, fluctuating oil prices, poor economic diversification (neglect of agriculture and industry), high inflation rates, low incomes, high cost of living, low economic growth rate, lack of domestic and foreign investments due to the weak investment environment, fragility of the private sector, and high dependence on the state **(** **Rasham et al., 2016** **: 384).**
  d) **Other reasons:** Natural disasters (drought, water scarcity, and the impact of climate change), migration, internal and external displacement that creates a gap in the labor market, social customs and traditions that limit the participation of some groups (such as women) in the labor market, the foreign occupation of Iraq, and the absence of strategic planning to address poverty comprehensively and long-term **(** **Al-Ghalibi, 2021** **: 82).**
2. **Economic Effects of Poverty in Iraq:** Poverty leaves deep economic effects in Iraq, most table the decline in domestic consumption, declining productivity, and weak savings and investment, as well as leading to the expansion of the informal economy and increasing the burdens on the public budget, which hinders the achievement of sustainable economic growth, including: **(** **Saeed and Daher, 2023** **: 215).**
  a) Reduced aggregate demand and domestic consumption due to weak purchasing power of households.
  b) Productivity declines due to poor education, malnutrition and lack of qualified competencies.
  c) The expansion of the informal economy and the use of the poor in precarious and unsecured jobs.
  d) Increased budget costs due to the growing need for social support programs and subsidies.
  e) Hindering economic growth because of lack of investment and a weak business environment.
  f ) Increasing inequality in the distribution of income, which leads to an economic and social gap between the classes of society.
  g) Poor savings and investment because most of the income of poor households goes only to necessary consumption.

**1-3: Theoretical Framework for Digital Transformation and Artificial Intelligence:**

Digital transformation and artificial intelligence are contemporary tools that contribute to supporting the effectiveness of macroeconomic policies and addressing poverty through the introduction of advanced technologies that contribute to improving economic management and enhancing the efficiency of targeting the most vulnerable groups, so digital transformation and artificial intelligence can be addressed and defined as follows:

1. **Digital Transformation:**

It is defined as the process of employing modern technologies to develop databases and strengthen economic information systems in a way that contributes to improving the management of public resources, increasing levels of transparency, and expanding access to information, and digital transformation provides accurate and wide-ranging data that supports the efficiency of fiscal, monetary, and trade policies, which enhances the ability of decision-makers to make decisions based on realistic data **(** **Mustafa and Noureddine, 2023** **: 53).**

2. **Artificial Intelligence:**

Artificial intelligence represents an advanced analytical dimension by relying on big data analysis techniques and predictive algorithms, which provides accurate tools for decision-makers to predict future economic trends, prioritize public spending, enable the design of economic and social programs that are more targeted to the poor, and enhance the ability of macroeconomic policies to achieve their goals of reducing poverty and promoting social justice **(** **Aldosari, 2020** **: 146).**

**1-4: The Theoretical Relationship between Macroeconomic Policy Tools and the Poverty Line according to Economic Logic:**

The logic of economic theory assumes that there is a direct relationship between the efficiency of macroeconomic policies and poverty levels, as a stable monetary policy reduces inflation and reduces income erosion, an efficient fiscal policy promotes social justice through redistribution and expansion of social spending, and a balanced trade policy creates a productive and operational environment that contributes to poverty reduction, and in turn, the imbalance or poor coordination of these policies exacerbates economic and social problems and increases poverty rates These relationships can be summarized as follows **(** **Baloul, 2009** **: 555):**

1. **The relationship between the broad money supply (M2) and the poverty line:** The broad money supply (M2) is inversely related to the poverty line, since an increase in the money supply usually leads to an expansion of economic activity, stimulates investment, creates new jobs, improves incomes, and thus reduces poverty rate **( Goshit and Longduut, 2016: 15).**
2. **The relationship between the inflation rate and the poverty line:** The inflation rate is directly related to the poverty line, because the higher the inflation rate, the lower the purchasing power of individuals, especially the poor and those with limited incomes, which leads to an increase in the poverty rate, and the impact of inflation on poverty is greater in rentier economies that depend on fixed cash income and limited resources **(** **Fawaz, 2021** **: 92).**
3. **The relationship between the average per capita foreign trade and the poverty line:** The average per capita foreign trade is inversely related to the poverty line, because if trade contributes to increasing productive opportunities, incomes, opening markets, and increasing exports from the studied imports, it creates job opportunities and improves incomes, which reduces the poverty rate **(** **Zaher and Ahmed, 2013** **: 484).**
4. **Relationship between government spending and poverty line:** Government spending is inversely related to the poverty line because directing spending towards education, health, infrastructure, and social transfers improves the standard of living and thus reduces the level of poverty.

## The second axis

### Standard modeling for the analysis of the effectiveness of macroeconomic policies in addressing poverty in Iraq for the period (2004-2024).

**2-1: Characterization of the Standard Model:**

The stage of characterization (formulation) of the Standard Model is one of the most important and difficult stages of building the economic model, as it requires the identification of the economic variables used in the Standard Model correctly and accurately by relying on economic theory and previous studies to transform the relationship between independent and dependent variables into mathematical equations and determine the direction and type of the relationship between those variables to build the Standard Model to analyze the effectiveness of macroeconomic policies in addressing poverty in Iraq, and the standard description stage of the used model includes two main steps, which are:

**First Step: Determining the Variables of the Standard Model Used**: Table 1 shows the time series data used in the standard side, as it includes the independent variables that represent the macroeconomic policy tools, which are four (broad money supply, inflation rate, average foreign trade per capita and government spending), and the dependent variable, which is the absolute poverty line index. It should be noted that the indicators of artificial intelligence and digital transformation for addressing poverty in Iraq are mostly qualitative, and the quantitative ones do not have available time series data for the period (2004–2024), The time series data for these variables can be presented as follows:

**Table 1.** Time series data of the search variables used in the standard model for the period (2004-2024).

Absolute poverty line (I.D)	Government spending (million dinars)	Average foreign trade per capita (one thousand dinars)	Inflation rate %	Broad money supply (million dinars)	Years
5	4	3	2	1	
48530	32117491	2358.4	26.8	12254000	2004
58436	26375175	3043.7	37.1	14684000	2005
88620	38806679	2974.5	53.1	21080000	2006
91411	39031232	2782.2	30.9	26956076	2007
103020	59403374	4162.4	12.7	34919675	2008
114173	55589721	3246.5	8.5	45437918	2009
117116	70134201	3666.2	2.5	60386086	2010
180700	78757667	4704.7	5.6	72177951	2011
247400	105139575	5470.5	6.1	75466360	2012
257859	119127556	5296.6	1.9	87679504	2013
263641	115937762	5234.9	2.2	90727801	2014
267616	82813611	3808.6	1.4	82595493	2015
188470	67067437	2972.1	0.6	88081993	2016
193891	75490115	3568.0	0.2	89441338	2017
188831	80873189	4641.5	0.4	92105401	2018
135928	111723523	4869.2	-0.2	103104122	2019
137299	76082445	3122.1	0.6	119906193	2020
101923	92241847	3419.5	6.1	139900000	2021
101654	57375038	5039.2	4.9	168291713	2022
100254	142435700	6778.4	4.4	170464256	2023
101632	150527650	5707.2	2.6	172732154	2024

Source: Prepared by the two researchers based on:

- Central Bank of Iraq, General Directorate of Statistics and Research, Annual Statistical Bulletins (2004-2024).

- Ministry of Planning, Central Bureau of Statistics, Directorate of National Accounts in different years.

- Ministry of Finance, Economic Department, General Budget Tables.

- Column (3) is calculated according to the following formula:

Average foreign trade per capita= $\frac{\text{Exports}+\text{Imports}}{\text{Population}}$ * 1000.

**Second Step: Specifictiom the Standard Model:**

The standard model for analyzing the effectiveness of macroeconomic policy tools in addressing poverty in Iraq can be formulated according to the following important relationship:

$$Y_{i}=a_{0}+b_{1}X_{1}+b_{2}X_{2}+b_{3}X_{3}+b_{4}X_{4}+u_{i}$$ (2)

Whereas:

Yi: Absolute poverty line, X1: Broad money supply, X2: Inflation rate, X3: Average foreign trade per capita, X4: Government spending.

**2-2: Results of the Time Series Stationary Test for the Research Variables according to the Unit Root Test:**

In order to make sure that the data of the time series of economic variables are more accurate and to detect the problem of the root of the unit, there are many tests that are used for this, and these tests are the Phelps Peron test, which has been employed in this research as the most accurate and reliable test in detecting the stillness, so the time series of the research variables must Passing this test for the purpose of determining the appropriate model in measuring and estimating the effectiveness of macroeconomic policies in addressing poverty in Iraq and Table 2 shows the results of the test:

**Table 2.** Results of the unit root test for stationary by test (PP) at level and first difference.

PP	Stationary of search variables time series at the original level					
	Variables	Y	X1	X2	X3	X4
With constant	t-Statistic	-2.2502	-2.8115	-2.9942	-1.3723	-3.1484
	Prob.	0.1908	0.1614	0.0399	0.5915	0.0272
	Result	n0	n0	**	n0	**
With Constant & Trend	t-Statistic	-2.7100	-3.3389	-2.9850	-2.0797	-3.2467
	Prob.	0.2358	0.2678	0.1432	0.5484	0.0833
	Result	n0	n0	n0	n0	*
Without Constant & Trend	t-Statistic	-2.2597	-3.0593	-3.0065	-1.2434	-3.0890
	Prob.	0.3239	0.1326	0.0031	0.1947	0.0024
	Result	n0	n0	***	n0	***

Stationary of search variables time series at the First difference						
	Variables	d(Y)	d(X1)	d(X2)	d(X3)	d(X4)
With constant	t-Statistic	-5.0706	-4.9499	-4.3532	-4.9004	-4.8449
	Prob.	0.0001	0.0001	0.0008	0.0001	0.0001
With Constant & Trend	Result	***	***	***	***	***
	t-Statistic	-5.0500	-4.9223	-4.3108	-4.8617	-4.8110
	Prob.	0.0005	0.0007	0.0052	0.0009	0.0011
	Result	***	***	***	***	***
Without Constant & Trend	t-Statistic	-5.0968	-4.9906	-4.3704	-4.9095	-4.8748
	Prob.	0.0000	0.0000	0.0000	0.0000	0.0000
	Result	***	***	***	***	***

Source: Prepared by the researchers based on the outputs of the statistical program (Eviews10).

- The sign (*) indicates that it is significant at the level of (10%), the sign (**) means its significance at the level of (5%), and the sign (***) means that it is significant at the level of (1%) respectively.

- n0: It indicates that it is insignificant.

It is clear from the results of Table 2 that not all of the research variables are static at the level except for the variables (X2, X4) that were static at the level according to the pp test, and since there are non-static variables at the zero level, the first difference should be taken and it is clear that all the variables have become static At the first difference of the variables’ data, where the calculated values of (t) were greater than their tabular value at the level of significance (1%), which means rejecting the hypothesis of nothingness (H _0_:B = 0) and accepting the alternative hypothesis (H _1_:B ≠ 0) that confirms that the time series in question are devoid of the root of the unit of stillness, and on this basis, it is preferable The use of the Autoregressive Distributed Lags Model (ARDL) as the research data are static at the level and the first difference, in addition to the number of observations (research sample) is small, and the quarterly data Are (84) observations for the period (2004-2024) were relied on using the standard program equation and adopting the linear formula The ARDL model in estimation to obtain constant linear relationships in which the estimators are interpreted as marginal salopes with respect to independent variables. Which takes the following formula:

$$∆Y_{t}=c+B_{1}Y_{t-1}+B_{2}X_{1t-1}+B_{3}X_{2t-1}+B_{4}X_{3t-1}+B_{5}X_{4t-1}+\sum_{i=1}^{p-1}\lambda_{1i}∆Y_{t-i}+\sum_{i=0}^{q_{1}-1}\lambda_{2i}∆X_{1t-i}+\sum_{i=0}^{q_{2}-1}\lambda_{3i}∆X_{2t-i}+\sum_{i=1}^{q3-1}\lambda_{4i}∆X_{3t-i}+\sum_{i=0}^{q_{4}-1}\lambda_{5i}∆X_{4t-i}+u_{t}$$ (3)

Y _t_: Dependent variable. X _K_: Independent variables. Δ : First differences.

C: Constant term. $u_{t}$ : Random error term. B: Long-run relationship parameters.

λ: Short-run relationship parameters at first differences *_._*

(P، q _1_، q _2_ … q _K_) Represent the optimal lag periods of the variables. (Y، X _1_، X _2_ … X _K_) Respectively.

Y _t_: Absolute poverty line, X _1_: Broad money supply, X _2_: Inflation rate, X _3_: Per capita foreign trade, X _4_: Government expenditure.

**2-3: Results of Estimating the Effectiveness of Macroeconomic Policies in Addressing Poverty in Iraq for the Period (2004-2024):**

**2.3.1: Preliminary estimate of the Poverty Model (ARDL):**

**Figure 1.**
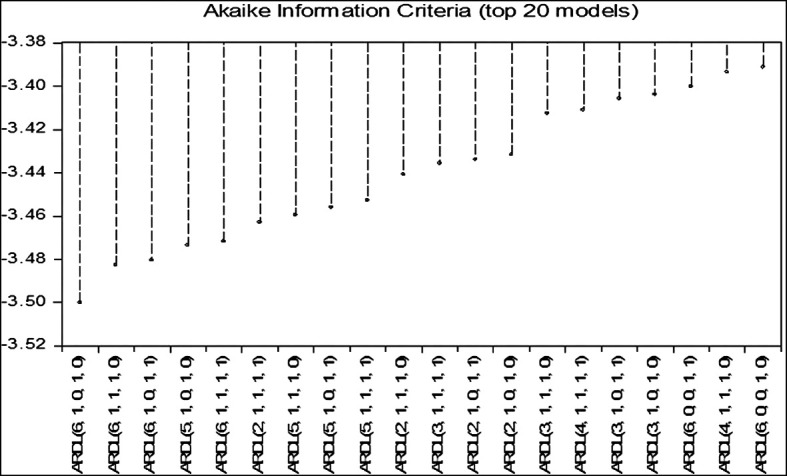
Optimal rank of the (ARDL) model according to the (AIC) standard.

Table 3 presents the results of the preliminary estimation of the Poverty Model (ARDL), which shows the relationship between the dependent variable (absolute poverty line) and the independent variables (macroeconomic policy tools) under discussion, as it is clear that the determination coefficient (R ^2^) was (0.94) and this value gives explanatory power is high for the studied standard model, i.e., the independent variables (macroeconomic policy tools) explain (94%) of the changes that occur in the dependent variable (absolute poverty line), and the remaining percentage (4%) represents the effect of other variables that were not included in the model, and the value of the test (F) statistic was (90.55) and it indicates The model used is significant in estimating the short- and long-term parameters, and that the optimal rank of the standard model selected according to the (ARDL) methodology is (0, 1, 0, 1, 6) based on the criteria of the optimal deceleration duration tests (HQ, BIC, AIC). The slowdown duration was chosen according to the (AIC) criterion because it represents the least value used for this criterion, and the Figure 1 shows the optimal rank of the (ARDL) model according to the (AIC) standard:

**Table 3.** Results of the preliminary estimate of the poverty model (ARDL) in Iraq.

Variable	Coefficient	Std. error	t-statistic	Prob.
Y(-1)	1.2675	0.1064	11.9071	0.0000
Y(-2)	-0.4146	0.1624	-2.5521	0.0134
Y(-3)	0.0000	0.1617	-0.0002	0.9999
Y(-4)	-0.3430	0.1667	-2.0573	0.0442
Y(-5)	0.5010	0.1634	3.0666	0.0033
Y(-6)	-0.1797	0.0994	-1.8072	0.0759
X1	-0.1159	0.0461	-2.5161	0.0147
X1(-1)	0.1179	0.0404	2.9178	0.0050
X2	0.0687	0.0350	1.9641	0.0543
X3	-0.9072	0.2195	-4.1323	0.0001
X3(-1)	0.9979	0.2184	4.5688	0.0000
X4	-0.0134	0.0079	-1.7048	0.0936
C	-0.0194	0.0081	-2.4053	0.0194
R-squared	0.9493	Mean dependent var		0.0140
Adjusted R-squared	0.9388	S.D. dependent var		0.1567
S.E. of regression	0.0387	Akaike info criterion		-3.5000
Sum squared resid	0.0870	Schwarz criterion		-3.0857
Log likelihood	137.2502	Hannan-Quinn criter.		-3.3353
F-statistic	90.5589	Durbin-Watson stat		2.0633
Prob(F-statistic)	0.0000			

Source: Prepared by the researchers based on the outputs of the statistical program (Eviews10).

**2.3.2: Results of the Bounds Test:**

To test the existence of a long-run equilibrium relationship between the independent variables (macroeconomic policy tools) and the dependent variable, using the statistics (F), Table 4 presents the results of the Cointegration test of the poverty model using the ARDL Boundary Test.

**Table 4.** Results of the cointegration test of the (ARDL) model for poverty according to the boundary test.

Test statistic	Value	K
F-statistic	8.492282	4
Critical Value Bounds		
Significance	Lower Bound	Upper Bound
10%	2.45	3.52
5%	2.86	4.01
2.50%	3.25	4.49
1%	3.74	5.06

Source: Prepared by the researchers based on the outputs of (Eviews10).

It is clear from Table 4 that the calculated value of the (F) statistic (8.492) which is higher than its tabular value at the level of (1%) significance, which means rejecting the null hypothesis (H _0_) that there is no long-term equilibrium relationship between the research variables, and accepting the alternative hypothesis (H _1_).) that there is a mutual complementarity relationship between the variables during the period under study, i.e. There is a long-term equilibrium relationship that goes from the explanatory variables (macroeconomic policy tools) to the dependent variable (absolute poverty line), which confirms the validity of the research hypothesis, which requires estimating the short- and long-term response and the error correction parameter (CointEq(-1)).

**2.3.3: Results of estimation of short- and long-term parameters and error correction parameters (CointEq(-1) for the (ARDL) model):**

After performing the bound test and verifying the existence of a long-term equilibrium relationship (co-integration) between the independent variables and the dependent variable, it is necessary to estimate the short-term and long-term parameters, in addition to the error correction parameter. The results in Table 5 indicate that there is a co-integration relationship between the independent variables This is confirmed by the error correction parameter of (-0.168) with a probability value of (prob=0.0001), which means that the error correction parameter achieved the negative and statistical significance conditions, i.e. (0.168) of short-term errors are automatically corrected within the unit of time to achieve equilibrium in the long run, and this result shows that the long-term is more responsive to changes in macroeconomic policy tools, which makes the error correction parameter statistically significant in the interpretation of the poverty model, so that the speed of adjustment (16%) is seen as evidence of the return of the model to equilibrium after any disruption. Poverty, represented by the absolute poverty line, requires about a year to reach its equilibrium value in the long term.

**Table 5.** Results of estimation of short- and long-term and error correction parameters of the ARDL model for poverty in Iraq.

Short-term parameters and error correction parameter				
Variable	Coefficient	Std. error	t-statistic	Prob
D(Y(-1))	0.4362	0.1001	4.3565	0.0001
D(Y(-2))	0.0216	0.0976	0.2216	0.8254
D(Y(-3))	0.0216	0.0974	0.2217	0.8253
D(Y(-4))	-0.3214	0.1031	-3.1169	0.0028
D(Y(-5))	0.1797	0.0994	1.8072	0.0759
D(X1)	0.1159	0.0461	2.5161	0.0147
D(X2)	0.0687	0.0350	1.9641	0.0543
D(X3)	0.9072	0.2195	4.1323	0.0001
D(X4)	-0.0134	0.0079	-1.7048	0.0936
CointEq(-1)	-0.1687	0.0397	-4.2527	0.0001

Cointeq = Y - (0.0114X1 + 0.4072X2 + 0.5376X3-0.0794X4-0.1150)				
Long-term parameters (Long Run Coefficients)				
Variable	Coefficient	Std. error	t-statistic	Prob.
X1	0.0114	0.1614	0.0706	0.0044
X2	0.4072	0.2224	1.8310	0.0022
X3	0.5376	0.3112	1.7277	0.0094
X4	-0.0794	0.0440	-1.8059	0.0061
C	-0.1150	0.0488	-2.3593	0.0017

Source: Prepared by the researchers based on the outputs of (Eviews10).

**2.3.4: Quality Assessment of the Estimated Standard Model:**

**First: Evaluating the quality of the model economically:**

**A: Evaluation of the parameters of the estimated model in the short and long term**: It is clear from the results in Table 5 related to measuring the effectiveness of macroeconomic policies in addressing poverty in Iraq for the period (2004-2024) and according to the following (ARDL) model:

1. The broad money supply coefficient (X1) indicates that there is a positive and significant effect of the broad money supply in Iraq on the (absolute poverty line) in the short and long term, as the value of the marginal slope of the poverty rate in the short term is (0.115) for the broad money supply, which means that the increase in the broad money supply in Iraq by one unit leads to an increase in the poverty rate by (0.115) in the short term, while the marginal slope of the poverty rate in the long run is (0.011) relative to the broad money supply, which means that an increase in the broad money supply in Iraq by one unit will lead to an increase in the poverty rate by (0.011) assuming that the other variables remain constant. This is inconsistent with the logic of economic theory, and this is since the monetary expansion in Iraq was not accompanied by an increase in domestic production but was reflected in the form of inflationary waves due to the economy’s dependence on imports and the weakness of the productive base.
2. The inflation rate coefficient (X2) indicates that there is a positive and significant effect of the inflation rate in Iraq on the (poverty rate) in the short and long term, as the value of the marginal slope of the poverty rate in the short term is (0.068) in relation to the inflation rate, which means that the increase in the inflation rate in Iraq by one unit ofIt leads to an increase in the poverty rate by (0.068) units in the short term, while the marginal slope of the absolute poverty line in the long term was (0.407) in relation to the inflation rate, which means that an increase in the inflation rate in Iraq by one unit will lead to an increase in the poverty rate by (0.407) units, assuming that the other variables remain constant. This is consistent with the logic of economic theory, since the high inflation rate leads to the erosion of the purchasing power of individuals, especially those with limited incomes, which reduces their ability to meet their basic needs, and in light of the weakness of wage policies and social protection networks in Iraq, the poor are more affected than others, so the direct relationship between inflation and poverty is economically logical, i.e., inflation expands the circle of poverty and deepens its severity.
3. The average per capita coefficient of foreign trade (X3) indicates that there is a positive and significant effect of the average per capita foreign trade in Iraq on the (poverty rate) in the short and long term, as the marginal slope of the poverty rate in the short term is (0.907) relative to the average per capita foreign trade This means that an increase in the average per capita foreign trade in Iraq by one unit leads to an increase in the poverty rate by (0.907) units in the short term, while the marginal slope of the absolute poverty line in the long run was (0.537) relative to the average per capita foreign trade, which means that an increase in the average per capita foreign trade by one unit will lead to an increase in the poverty rate in Iraq by (0.537). This is not in line with the logic of economic theory, as theoretically, the rise in the average per capita foreign trade is supposed to improve living standards and reduce poverty, but the reality in Iraq shows that the revenues of trade, especially oil, are not reflected fairly on all groups, especially the poor ones, due to poor distribution and weak social policies.
4. The government expenditure coefficient (X4) indicates that there is an adverse and significant effect of government spending in Iraq on the absolute poverty line in the short and long term, as the value of the marginal slope of the absolute poverty line in the short term is (-0.013) for government spending, which means that the increase in government expenditure In Iraq, by one unit leads to a reduction in the poverty rate by (0.013) In the short term, Whil the marginal slope of the absolute poverty line in the long term was (-0.079) for government expenditures, which means that an increase in government spending in Iraq by one unit will lead to a reduction in the poverty rate by (0.079) units, assuming that other factors are stable, This is Comsistpemt with the logic of economic theory, since increasing government spending, especially in the areas of basic services such as education, health, and social protection networks, contributes to improving the standard of living of individuals and reducing the economic gap, and directing spending towards investment and infrastructure creates new job opportunities and increases national income, so the inverse relationship between government spending and poverty is logical in the economic situation of Iraq.

**B. Measuring the Effectiveness of Macroeconomic Policies in Affecting Poverty in Iraq:**

Macroeconomic policymakers focus on estimating long-term parameters to show the effectiveness of explanatory variables in the dependent variable, because these estimates reflect the total effect (direct and indirect) of the change in explanatory variables, whether external or internal, time-delayed in the dependent variable, while the short-term parameters are concerned with measuring the direct effect only, and Table 6 shows the effectiveness of independent variables in influencing the absolute poverty line in Iraq.

**Table 6.** The effectiveness of macroeconomic policies in influencing poverty in Iraq.

Ratio of short-run to long-run parameter effectiveness (%)	Effectiveness of long-run parameters (total effect)	Effectiveness of short-run parameters (direct effect)	Variables
1017	0.0114	0.1159	x1
17	0.4072	0.0687	x2
169	0.5376	0.9072	x3
17	-0.0794	-0.0134	x4

Source: Prepared by the researchers based on the results in Table (5).

Table 6 shows that the highest effectiveness rate in changes in the absolute poverty line in Iraq is due to the broad money supply (monetary policy), as it reached in the short term (1017%) of the total impact, followed by the effectiveness of the average per capita foreign trade, which reached its effectiveness in the poverty rate in the short term (169%) of the total impact, while the effectiveness rate reached 169% of the total impact Both the inflation rate variable and government expenditure in the absolute poverty line in the short term (17%) of the total impact were the lowest effective compared to the other variables. It is inferred from this that monetary policy is the most effective in influencing poverty in Iraq, which confirms the validity of the research hypothesis.

**Second: Evaluation of the Statistical Quality of the Estimated (ARDL) Model**: Table 7 shows the descriptive statistical indicators that confirm the complete validity of the statistically estimated model, as all the independent variables were significant according to the (t) test in the short and long term, as well as the increase in the value of the corrected determination coefficient which reached ( ${\bar{R}}^{2}=0.93$ ) which explains that the estimated model explains (93%) of the changes in the dependent variable (poverty rate), and that the remaining percentage (7%) is a effect of other variables that were not included in the model, and the value of the statistic (F) reached (90.55), which confirms the significance of the estimated model as a whole at a significant level of less than (1%), as well as the decrease in the value of the standard error (S.e) which reached (0.038).

**Table 7.** Statistical indicators of the ARDL model for the estimated poverty line.

R-squared	0.9493
Adjusted R-squared	0.9388
S.E. of regression	0.0387
Sum squared resid	0.0870
Log likelihood	137.2502
F-statistic	90.5589
Prob (F-statistic)	0.0000
Durbin-Watson stat	2.0633

Source: Prepared by the researchers based on the outputs of the statistical program (Eviews10).

**Figure 2.**
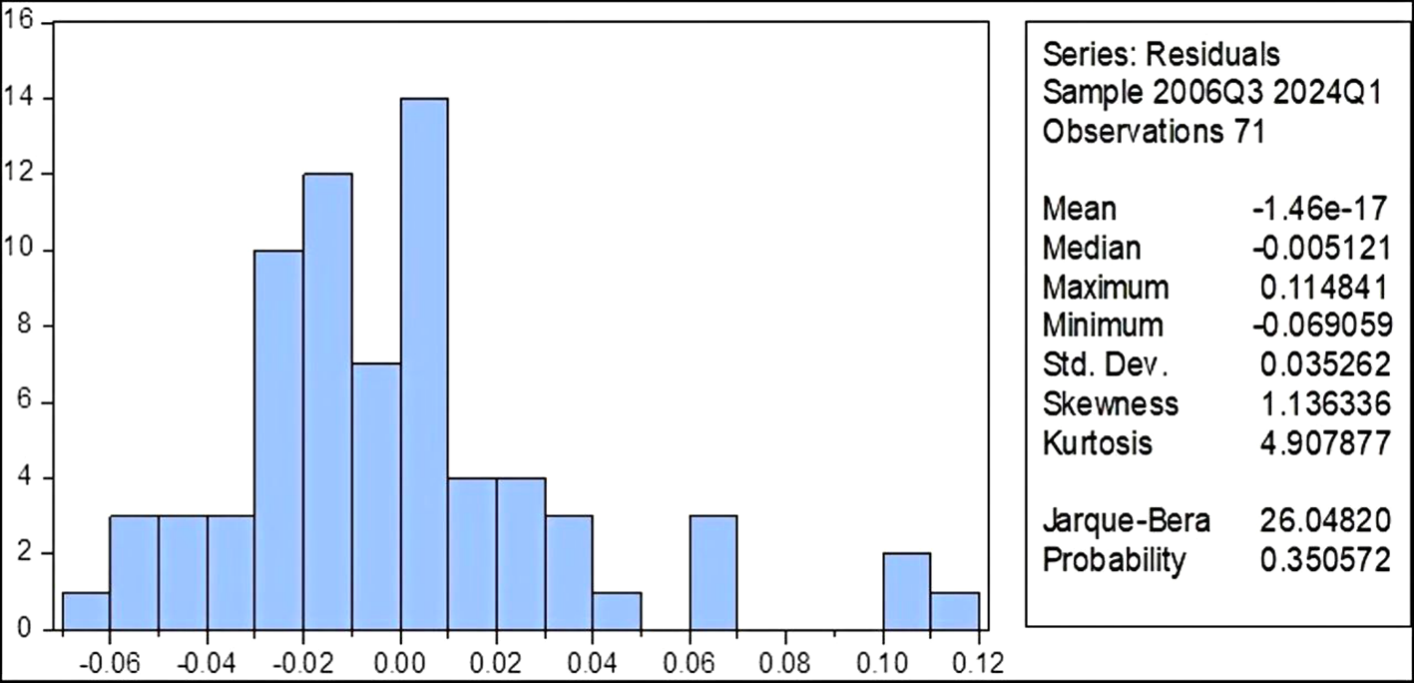
Test the normal distribution of the residuals of the estimated poverty model in Iraq.

**Third: Evaluation of the quality of the model in terms of the standard:** After estimating the parameters of the standard model for the short and long term relationship, and for the purpose of confirming the quality of the standard model used in measuring the effectiveness of macroeconomic policies in addressing poverty in Iraq and free from all standard problems, the following diagnostic tests are required:

1. **Jarque-Bera test for random errors**: Figure 2 shows that the random errors of the estimated model follow the Normal distribution, since the value of the JB test was (26.048) and a probability value of (0.350), which confirms the possibility of accepting the null hypothesis that random errors are Normal distributed.
2. **Auto correlation test:** It is clear from Table 8 that shows the results of the Auto correlation test based on the LaGrange multiplier serial correlation test (BGLM), which is the most appropriate test in detecting the existence of self-correlation between the random variable series data, the test shows that the model does not suffer from the problem of sequential Auto correlation because the probability value associated with both the (F) test Chi-square was greater than the significance level (5%), where the probability value of the statistic (F) was (0.6311), and the probability value of the chi-square statistic was (0.5606), which means that Accepted the hypothesis (H _0_) that the estimated model (absolute poverty line) is free of the problem of sequential autocorrelation.This is reinforced by the D-W statistic value of (2.0633).
3. **Variance Homogeneity Test for Random Errors:** It is clear from Table 9 that the results of the Variance Homogeneity Test for Random Errors shows that the poverty rate model under study does not suffer from the problem of Heteroskedasticity because the calculated value of (F) statistic was (1.3815) with a non-significant probability value of (0.2441), which means accepting the nullity hypothesis that the random error limit variation in the estimated model is homogeneous.
4. **Ramsey RESET Test**: Table 10 shows the validity of the deli shape of the estimated model, as it is clear that the calculated statistic (F) was (5.4884) with an insignificant probability value of (0.5227), in addition to the calculated (t) statistic (2.3427) with an insignificant probability value of (0.5227). This means accepting the nullity hypothesis that the description of the delusional figure used in the estimating model of poverty is correct.
5. **Results of the Structural Stability Test of the Estimated ARDL Coefficients:** In order to ensure that the data used in estimating the (poverty rate) model are free of structural changes and the extent of stability and consistency of the long-term parameters with the estimated short-term parameters, therefore, the cumulative sum of Recursive residual test (Cusum) and the cumulative sum of Squares Recursive residual test (Cusum-SQ) were used. if the graph of the two tests falls within the critical threshold at a significant level (5%), Will accept the null hypothesis that all estimated parameters are structurally stable as shown in the following figure:

**Table 8.** Results of the auto correlation test (LM) for the estimated poverty model.

Breusch-Godfrey serial correlation LM test			
F-statistic	0.4641	Prob. F(2، 56)	0.6311
Obs*R-squared	1.1577	Prob. Chi-Square(2)	0.5606

Source: Prepared by the researchers based on the outputs of the statistical program (Eviews10).

**Table 9.** Results of the Heteroskedasticity Test (ARCH) of the estimated poverty model.

Heteroskedasticity Test: ARCH			
F-statistic	1.3815	Prob. F(1، 60)	0.2441
Obs*R-squared	6.8138	Prob. Chi-Square(5)	0.2349

Source: Prepared by the researchers based on the outputs of the statistical program (Eviews10).

**Table 10.** Results of the validity test of the correctness of the functional form of the estimated poverty model.

Ramsey RESET test			
Equation: UNTITLED			
Omitted variables: Squares of fitted values			
Test	Value	Df	Probability
t-statistic	2.3427	57	0.5227
F-statistic	5.4884	(1، 57)	0.5227

Source: Prepared by the two researchers based on the outputs of the statistical program (Eviews10).

**Figure 3.**
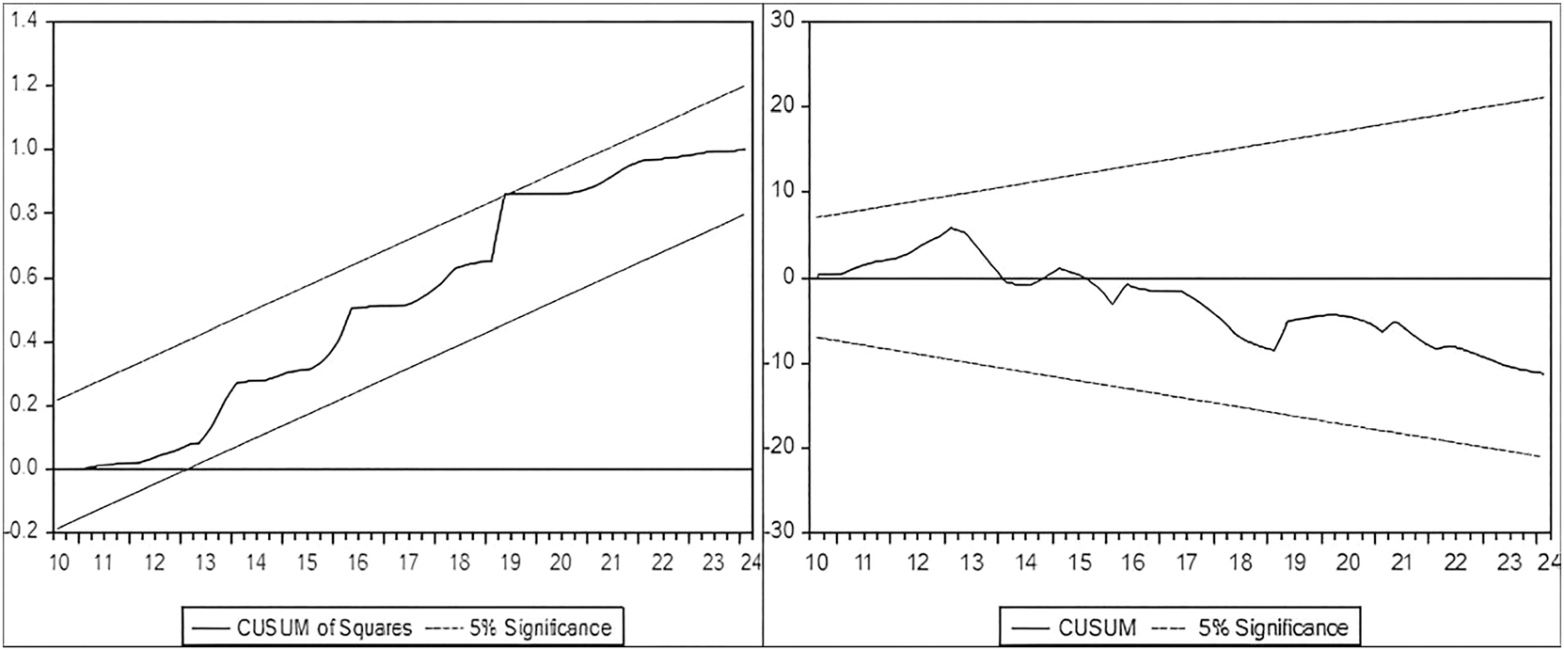
Testing the structural stability of the ARDL poverty model coefficients in Iraq.

It is clear from Figure 3 that the graph of the above two tests was within the critical limits (upper and lower limits) at the level of significance (5%) and varies around the value of zero, and it is concluded from these two tests that there is stability and consistency in the model’s estimates between the results of the short and long term of the model (ARDL) of the poverty rate in Iraq during the research period.

**Figure 4.**
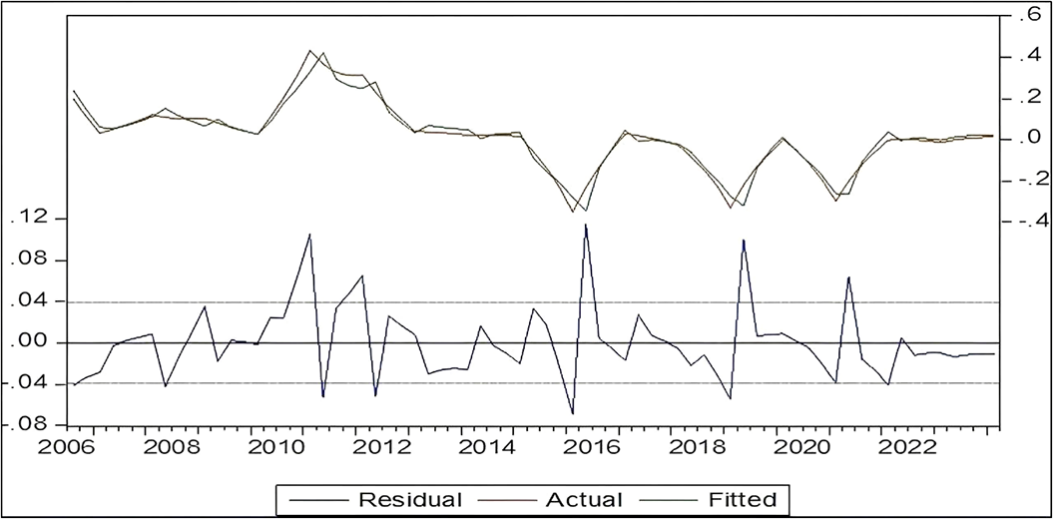
Results of the interpretive ability test of the (ARDL) model for poverty in Iraq.

6. **Results of the Interpretive Ability Test of the Estimated (ARDL) Model:** It is clear from Figure 4 the results of the Interpretive Ability Test of the (ARDL) model of the estimated real and estimated values as well as the values of the random error limit, which shows that there is a convergence between the predictive values and the real values, and this indicates that the estimated (poverty rate) model is characterized by a high explanatory ability.

## The Third Axis: Conclusions and Recommendations

### 3.1: Conclusions

1. The results of estimating the standard model proved that there is a long-term equilibrium relationship between the independent variables (macroeconomic policy tools) and the dependent variable (absolute poverty line) using the Bound Test methodology of the (ARDL) model, where the value of the statistic (F) was Greater than the critical values of the upper and lower limits at a significant level (1%).
2. The results of the standard analysis proved that there is a significant positive effect of the broad money supply (X1) on the absolute poverty line, which does not correspond to the logic of economic theory, and the results also showed that there is a significant positive effect of the inflation rate (X2) on the absolute poverty line, which is consistent with the logic of economic theory.
3. The results of the standard analysis proved that there is a significant positive effect of the average per capita foreign trade (X3) on the absolute poverty line, which does not match the logic of economic theory, while the results showed that there is a negative and significant response between government expenditure (X4) and the absolute poverty line, which is consistent with the logic of economic theory.
4. The effectiveness of each of the variables (broad money supply, average foreign trade per capita, inflation rate, and government spending) in the absolute poverty line in the short term (1017%, 169%, 17%, 17%). From the total effect, respectively, as the highest effectiveness rate in changes in the poverty rate was due to The broad cash supply reached in the short term (1017%). From the total impact, indicating that monetary policy is the most effective in addressing poverty in Iraq during the research period.
5. The monetary policy in Iraq during the period (2004-2024) was more effective in influencing poverty rates compared to the fiscal and trade policies, but this effect was negative as it contributed to the increase in inflation rates and the decline in the purchasing power of households, especially the low-income groups, due to the fact that economic theory assumes a stable economic environment and strong financial institutions capable of transferring the impact of monetary policy tools in a balanced manner, while the Iraqi economy was characterized by its weak production structures and its adoption The near-absolute impact of oil revenues, as well as political and security instability, combined with these factors, have distorted the transmission mechanism of monetary policy and made it a tool that exacerbates rather than reduces poverty, In addition to minimizing the impact of artificial intelligence and digital transformation technologies in contributing to poverty alleviation in Iraq.
6. Integrating digital transformation and artificial intelligence into the formulation of macroeconomic policies is not merely a technological update but a strategic revolution that reshapes the entire decision-making mechanisms. The limited impact of public spending on poverty reduction fades in the face of the power of the digital economy, which transforms data into smart, fair, and effective policies, making poverty alleviation a tangible outcome of advanced economic intelligence that anticipates crises and creates opportunities for sustainable development.
7. The estimated standard model (absolute poverty line) passed all statistical and standard tests, as tests of the model’s suitability proved that the model was free of standard and statistical problems.

### 3.2: Recommendations

1. Since high monetary expansion leads to higher inflation and higher poverty rates, flexible monetary policies must be put in place that curb hyperinflation and encourage fiscal stability, considering the balance between government financing for growth and poverty reduction.
2. Combat inflation and protect the purchasing power of the poor by using monetary and fiscal policy tools such as interest rates and control of the money supply while providing direct support to the poor to reduce the impact of rising prices on their standard of living.
3. Promoting foreign trade and increasing its per capita, as encouraging exports and diversifying sources of foreign trade contribute to increasing per capita income and improving income distribution, as well as the need to facilitate investment in productive sectors and support local companies to achieve higher added value.
4. Improve the efficiency of government spending by focusing on shifting spending from a consumption pattern to investment in infrastructure and basic social services with strict control over corruption to ensure that financial resources reach the actual beneficiaries and contribute to effectively reducing poverty.
5. Develop comprehensive strategies to address poverty by combining monetary, fiscal and trade policies and creating a state of integration to ensure a balance between economic stability and sustainable growth with a focus on social programs that support education, health, and employment for the poor.
6. Developing a system of monitoring and analyzing economic data through the establishment of periodic monitoring mechanisms for poverty, inflation, money supply and trade indicators to assess the effectiveness of macroeconomic policies and adjust them quickly according to economic developments.
7. Adopting a comprehensive national strategy to integrate digital transformation and artificial intelligence technologies into the design and implementation of macroeconomic policies to address poverty in Iraq, through the establishment of a National Council for Digital Transformation and Artificial Intelligence within the Ministry of Planning, responsible for coordinating among ministries and government institutions, activating electronic linkage via unified and reliable databases that enable data and information exchange, involving the private sector in developing digital infrastructure, developing and enhancing financial inclusion platforms targeting the most vulnerable groups, making intensive investments in digital infrastructure, training and qualifying national cadres, and strengthening research partnerships with local and international institutions, as the implementation of this strategy represents a crucial entry point for achieving economic justice, effectively reducing poverty, and ensuring the efficiency and sustainability of the national economy in the long term.

## Ethical approval

This study received ethical approval from the Scientific Research Ethics Committee of the Scientific Affairs Unit at the University of Fallujah. All research procedures were conducted in accordance with the approved ethical standards, The ethical approval was formally granted under the reference code **UOF.HUM.2025.001**.

## Data Availability

The data supporting the findings of this study are openly available in [Zenodo] at [ https://doi.org/10.5281/zenodo.18405756], ( Al Mihimdy & Attia, 2026) under the Creative Commons Attribution 4.0 International (CC BY 4.0) license. The datasets used and analyzed in this study are publicly available from official Iraqi sources, including: Iraqi Ministry of Planning – www.mop.gov.iq Iraqi Ministry of Finance – www.mof.gov.iq Central Bank of Iraq – www.cbi.iq The datasets analyzed in this study are publicly available from official Iraqi sources, including the Ministry of Planning, the Ministry of Finance, and the Central Bank of Iraq. These include broad money supply, inflation rate, per capita foreign trade, government expenditure, and the absolute poverty line. All data are freely accessible and can be reused under an open licence. Repository name: [Analyzing the Effectiveness of Macroeconomic Policies in Addressing Poverty in Iraq: An Econometric Study considering Digital Transformation and Artificial Intelligence for the Period (2004–2024)]. [ https://doi.org/10.5281/zenodo.18405756]. ( Al Mihimdy & Attia, 2026) The project contains the following underlying data: [Search data.xlsx] (Raw numerical data used for the main statistical analysis). No extended data are associated with this article. Data are available under the terms of the Creative Commons Attribution 4.0 International (CC BY 4.0). This study is a standard statistical investigation. No CONSORT or ARRIVE checklists are required as the study does not involve clinical trials or animal experiments.
