# Supplementary material for: Analyzing the Effectiveness of Macroeconomic Policies in Addressing Poverty in Iraq: An Econometric Study considering Digital Transformation and Artificial Intelligence for the Period (2004–2024)
Source: F1000Res. 2026 Feb 6;15:209. [Version 1] doi: 10.12688/f1000research.174407.1 (PMC12972709; doi:10.12688/f1000research.174407.1)
Supplement: Peer review history

# Reviewer report: Reviewer response for version 1

**DOI:** 10.5256/f1000research.192303.r456769

**Published:** 2026-03-09

**Authors:** Nora Amelda Rizal

**Affiliations:** 1 Telkom University, Bandung, Indonesia

Dear Authors, 

 First of all, congratulations on your research, and thank you for allowing me to read your work. The topic is interesting, not only well-targeted in its implementation but also highly relevant for addressing poverty in a complex economic landscape like Iraq. Integrating macroeconomic policy evaluation with modern elements like digital transformation and AI adds a new dimension to this research.

 I've looked over your research draft about how well macroeconomic policies work in Iraq very carefully. The work that went into the ARDL modelling is clear, and the use of modern topics like Digital Transformation and AI shows a strong desire to bring Iraqi economic discourse up to date.

 However, allow me to make some comments on this manuscript. There are several points that I feel would further strengthen the argument for the importance of this research and a more precise methodology. 

 1. I noticed that the theoretical framework in Axis 1 would benefit from a more systematic approach, such as the PRISMA or SLR (Systematic Literature Review) methodology. Currently, the literature doesn’t provide a strong, synthesized gap analysis; it is more like just definitions. Strengthening the narrative bridge between your chosen variables and the specific socio-economic context of Iraq would more effectively justify the conceptual framework.  Also,  you must clarify why certain variables were selected for the ARDL model while others (like AI) remain strictly theoretical.

 2. The paper strongly suggests that there will be an analysis of Digital Transformation and AI. However, there is a clear disconnect because these factors don't fit into the empirical model (Axis 2) because the data is known to be limited. To maintain academic rigor, it would be beneficial to reframe these technologies as essential elements of the proposed solution or policy framework in the recommendations, rather than representing them as primary variables for statistical analysis.

 3. The ARDL model is a deductive tool for testing established macroeconomic theories against data. Therefore, I recommend improving the definition of the "blended approach." If you don't collect any qualitative primary data separately (like through interviews or Delphi methods), calling the study "Deductive Quantitative" would better describe the advanced econometric work you've done.

 4. The ARDL results show a strong link between Money Supply X1 and levels of poverty. You also say this to show that Monetary Policy is the "most effective" because it has a 1017% effect (conclusion 4).  But because the correlation is positive (meaning it makes poverty worse), this should be seen as a major failure or bad effect instead of an effective one. If a policy consistently moves the variable away from the desired target (reducing poverty), it can't be called effective.

 5. Conclusion no 6 should be more related to your quantitative results. The ARDL results could show that "traditional" tools haven't worked, making the Digital Transformation you suggest in the last section logically necessary.

 You should also explicitly link how AI can mitigate the failures in your ARDL results to strengthen the 'AI Strategic Revolution' in your conclusion. Instead of treating AI as a general concept, you could frame it as a targeted solution, such as suggesting that AI-driven Predictive Analytics could improve forecasting to prevent the 1017% impact of monetary expansion or that Machine Learning algorithms could optimize Government Expenditure (X3) to reduce poverty. This method turns the AI narrative into a data-driven solution to your study's empirical issues."

 7. Last issue, it appears the manuscript may be trapped by the term "Effective" used in the initial hypothesis. I suggest using more neutral terms, such as "Significant Impact" or "Influential Factor." This allows you to stay true to your empirical findings (where the impact is significant but the outcome is negative) without compromising the paper's credibility.

 I hope my feedback proves useful in refining your research. I look forward to seeing the improved version of this manuscript

 Best Regards.

Is the work clearly and accurately presented and does it cite the current literature?

Yes

If applicable, is the statistical analysis and its interpretation appropriate?

Yes

Are all the source data underlying the results available to ensure full reproducibility?

Yes

Is the study design appropriate and is the work technically sound?

Yes

Are the conclusions drawn adequately supported by the results?

Yes

Are sufficient details of methods and analysis provided to allow replication by others?

Yes

Reviewer Expertise:

Artificial Intelligence in Business, Financial Market, Mathematical Finance, Behavioral Finance, Investment & Portfolio

I confirm that I have read this submission and believe that I have an appropriate level of expertise to confirm that it is of an acceptable scientific standard.

# Reviewer report: Reviewer response for version 1

**DOI:** 10.5256/f1000research.192303.r461342

**Published:** 2026-03-02

**Authors:** Antonio Sánchez Bayón

**Affiliations:** 1 Universidad Rey Juan Carlos, Móstoles, Spain

Dear authors,

 Congratulations for your effort. Please, the following comments are some suggestions to improve your paper.

 Your methodological point is ok, but maybe you could improve the theoretical framework. Perhaps it would be appropriate to clarify the schools of thought used and not just particular papers. Thus, on one side there would be the interventionists or degrowthers (pro state control -including digitalization-, coercive central planning and supervised growth, i.e. new and post-Keynesians), on the other side the liberals or developers (pro social and market change, entrepreneurship and digitalization, i.e. Austrian Economics, New-institutional Economics). Examples of readings that clarify this classification, in addition to addressing complementary problems to check in your paper (secondary round effects of transformation according to each school of thought: cobra effect, readjustment effect, inflation, etc.). Please, see [Reference 1]

 Please, revise the figures/tables (please, include source, v.g. "own elaboration").

 Please, review the edition style.

 Good job and best regards.

Is the work clearly and accurately presented and does it cite the current literature?

Yes

If applicable, is the statistical analysis and its interpretation appropriate?

Yes

Are all the source data underlying the results available to ensure full reproducibility?

Yes

Is the study design appropriate and is the work technically sound?

Yes

Are the conclusions drawn adequately supported by the results?

Yes

Are sufficient details of methods and analysis provided to allow replication by others?

Yes

Reviewer Expertise:

Comparative Political Economy & Socioeconomics (i.e. Austrian Economics, Ancaps, Cultural & Neoinstitutional Economics), with special attention to digitalization & Wellbeing Economics.

I confirm that I have read this submission and believe that I have an appropriate level of expertise to confirm that it is of an acceptable scientific standard.

## References

[1] : Public management of digitalization into the Spanish tourism services: a heterodox analysis. *Review of Managerial Science* .2025;19(7) : 10.1007/s11846-024-00753-1 2235-2253 10.1007/s11846-024-00753-1
